# Global Distribution of Bartonella Infections in Domestic Bovine and Characterization of *Bartonella bovis* Strains Using Multi-Locus Sequence Typing

**DOI:** 10.1371/journal.pone.0080894

**Published:** 2013-11-21

**Authors:** Ying Bai, Lile Malania, Danilo Alvarez Castillo, David Moran, Sumalee Boonmar, Aran Chanlun, Fanan Suksawat, Soichi Maruyama, Darryn Knobel, Michael Kosoy

**Affiliations:** 1 Bacterial Disease Branch, Division of Vector-Borne Disease, Centers for Disease Control and Prevention, Fort Collins, Colorado, United States of America; 2 National Center for Disease Control and Public Health, Tbilisi, Republic of Georgia; 3 Centro de Estudios en Salud, Universidad del Valle de Guatemala, Guatemala, Guatemala; 4 International Emerging Infectious Program, Thailand MOPH-US CDC Collaboration, Nonthaburi, Thailand; 5 Khon Kaen University, Khon Kaen, Thailand; 6 Department of Veterinary Medicine, College of Bioresource Science, Nihon University, Kanagawa, Japan; 7 Department of Veterinary Tropical Diseases, University of Pretoria, Pretoria, South Africa; 8 Kenya Medical Research Institute/Centers for Disease Control and Prevention Public Health Collaboration, Kisumu, Kenya; Technion-Israel Institute of Technology Haifa 32000 Israel, Israel

## Abstract

*Bartonella bovis* is commonly detected in cattle. One *B. bovis* strain was recently isolated from a cow with endocarditis in the USA, suggesting its role as an animal pathogen. In the present study, we investigated bartonella infections in 893 cattle from five countries (Kenya, Thailand, Japan, Georgia, and Guatemala) and 103 water buffaloes from Thailand to compare the prevalence of the infection among different regions and different bovid hosts. We developed a multi-locus sequence typing (MLST) scheme based on nine loci (16S rRNA, *gltA*, *ftsZ*, *groEL*, *nuoG*, *ribC*, *rpoB*, *ssrA*, and ITS) to compare genetic divergence of *B. bovis* strains, including 26 representatives from the present study and two previously described reference strains (one from French cows and another from a cow with endocarditis in the USA). Bartonella bacteria were cultured in 6.8% (7/103) of water buffaloes from Thailand; all were *B. bovis*. The prevalence of bartonella infections in cattle varied tremendously across the investigated regions. In Japan, Kenya, and the Mestia district of Georgia, cattle were free from the infection; in Thailand, Guatemala, and the Dusheti and Marneuli districts of Georgia, cattle were infected with prevalences of 10–90%. The *Bartonella* isolates from cattle belonged to three species: *B. bovis* (n=165), *B. chomelii* (n=9), and *B. schoenbuchensis* (n=1), with the latter two species found in Georgia only. MLST analysis suggested genetic variations among the 28 analyzed *B. bovis* strains, which fall into 3 lineages (I, II, and III). Lineages I and II were found in cattle while lineage III was restricted to water buffaloes. The majority of strains (17/28), together with the strain causing endocarditis in a cow in the USA, belonged to lineage I. Further investigations are needed to determine whether *B. bovis* causes disease in bovids.

## Introduction

Found throughout much of the world, cattle are the most common type of large domesticated ungulate. They comprise hundreds of breeds that are recognized worldwide. Previously described as two separate subspecies (*Bos taurus* subsp. *taurus* and *B. taurus* subsp. *indicus*), all cattle belong to the sub-tribe Bovina under tribe Bovini according to a very recent classification based on multiple autosomal introns [1]. Water buffaloes (*Bubalus bubalis*) resemble cattle in many characteristics but belong to Bubalina, another sub-tribe under Bovini [1]. These animals are agriculturally and economically important to humans as they are widely used for a variety of purposes, such as meat, dairy products, and draught power. However, a variety of infections in cattle, such as bovine spongiform encephalopathy, foot-and-mouth diseases, brucellosis, anthrax, infectious endocarditis, and many others, have caused either economical loss of these animals or big public health concerns as some of these infections are transmissible to humans and potentially can cause death. 

The genus *Bartonella* contains *B. bovis* and many other species and/or subspecies. These fastidious Gram-negative bacteria infect and persist in mammalian erythrocytes and endothelial cells and are found in a wide range of wild and domesticated mammals, including rodents, insectivores, carnivores, ungulates, and others. A number of *Bartonella* species have been associated with human illnesses and are responsible for a growing spectrum of emerging diseases, including endocarditis [2-10]. Knowledge of the transmission of Bartonella bacteria between mammalian hosts is incomplete. However, hematophagous arthropods, such as fleas, flies, lice, mites, and ticks, have been found naturally infected and are frequently implicated in transmitting *Bartonella* species [11-16]. 

Infection with *Bartonella* spp. (mainly *B. bovis*) in cattle has been reported in a number of studies [17-20], with *B. bovis* originally described in cows from France [21]. For water buffaloes, there are no previous reports of infections with *Bartonella* spp. The prevalence of *B. bovis* in cattle is generally high but varies widely across studies from different countries, i.e., 50-89%, 70%, 36%, 24%, and 20%, in the USA, French Guyanna, France, Italy, and West Africa, respectively [18-23]. In a very recently study from Poland, the prevalence of *B. bovis* in cattle was much lower (6.8%) [24]. In addition to *B. bovis*, two more *Bartonella* spp. (*B. chomelii* and *B. schenobuensis*) are occasionally found in cattle [17,25]. Although *Bartonella* spp. do not usually cause clinical signs in cattle, *B. bovis* is associated with bovine endocarditis [26,27]. Understanding whether any specific genetic variations are responsible for the pathogenic outcome in animals and people is important and requires separate investigation. 

In the present study, we first investigated the prevalence of *Bartonella* spp. in cattle from five countries across the world (Kenya, Thailand, Japan, Georgia, and Guatemala) and in water buffaloes from Thailand. Following that, we characterized the *B. bovis* strains obtained from the present study and from reference collections (France and USA), and then compared the genetic difference among these strains. We employed multi-locus sequence typing (MLST) that is based on comparison of nucleotide sequences derived from multiple loci. This approach has been applied to study genetic diversity of different agents, including *Bartonella* spp. [28-30]. It has been shown to provide high discriminatory power in epidemiological and genetic analysis of strain populations while retaining signatures of longer-term evolutionary relationships or clonal stability. This in turn can help to enhance our understanding of population structure of the bacteria and the relationships between sequence type and animal host. Our aims were: (1) to assess the apparent prevalence of bartonella culture-positive infections in cattle and water buffaloes from different countries; (2) to identify *Bartonella* spp. in positive samples using the citrate synthase gene (*glt*A); and (3) to develop a MLST scheme for *B. bovis*, and to validate this scheme against a diverse sample of strains representing each country in this study to allow comparison of their sequence diversity. 

## Materials and Methods

### Ethics Statement

Specimen collection from cattle in Kenya was approved by the Institutional Animal Care and Use Committees (IACUC) of the Kenya Medical Research Institute (protocol no. 1191) and CDC (1562BRETBDX). Sampling cattle does not require IACUC approval in Guatemala. There was no IACUC or ethics committee in Georgia, Thailand, and Japan at the time of the conducted field study. 

Cattle samples were collected in private ranches in all sites. There was no specific permission required. Verbal or written informed consent was obtained from all animal owners before specimen collection. Sample collection was done by veterinarians, experienced field technicians, and trained animal health assistants. All animal procedures were conducted in compliance with the Animal Welfare Act and other Federal statutes and regulations relating to animals and experiments involving animals and adheres to principles stated in the *Guide for the Care and Use of Laboratory Animals* (National Research Council [NRC] Publication. Samples from Guatemala and Thailand were obtained specifically for this study; and samples from Kenya, Japan, and Republic of Georgia were obtained as part of routine care. Three milliliters of blood were collected from each animal. 

### Study sites, sample collection, and reference strains

Whole blood samples were collected from healthy cattle and water buffaloes from five countries during different time periods (Table 1). Samples collected included (1) 40 cattle from Chachoeng Sao Province of Thailand and 103 water buffaloes from Chachoeng Sao, Nakhon Pathom, and Khon Kaen provinces of Thailand; (2) 305 cattle from Chiba, Hokkaido, Kagoshima, Kanagawa, and Okinawa prefectures of Japan; (3) 159 cattle from Dusheti, Marneuli, and Mestia districts of Republic of Georgia; (4) 389 cattle from Alta Verapaz, Huehuetenango, Izabal, Quetzaltenango, Quiche, and Retalhuleu departments of Guatemala; and (5) 221 cattle from Nyanza Province of Kenya. Blood samples were kept at -20° C or lower temperature until tested. All samples were sent to the Bartonella Laboratory, CDC in Fort Collins, Colorado, USA for bartonella testing, except samples from Japan, which were tested in Laboratory of Veterinary Public Health, Nihon University, Kanagawa, Japan using analogous culturing techniques [17].

**Table 1:** Distribution of bartonella infection in cattle from different geographic regions.

Country	Region	# Tested	# Positive	Prevalence (%)	Collection date
Thailand	Chachoeng Sao Province	40	4	10	September, 2008
Japan	Chiba Prefecture	37	0	0	July, 2000 - February, 2005
	Hokkaido Prefecture	52	0	0	
	Kagoshima Prefecture	139	0	0	
	Kanagawa Prefecture	57	0	0	
	Okinawa Prefecture	20	0	0	
	*total*	*305*	*0*	*0*	
Georgia	Dusheti District	100	73	73	May - June, 2010
	Marneuli District	20	18	90	
	Mestia District	39	0	0	
	*total*	*159*	*91*	*57.2*	
Guatemala	Alta Verapaz Department	60	15	25	April - December, 2011
	Huehuetenango Department	110	12	10.9	
	Izabal Department	60	6	10	
	Quetzaltenango Department	37	18	48.6	
	Quiche Department	62	22	35.5	
	Retalhuleu Department	60	7	11.7	
	*total*	*389*	*80*	*20.6*	
Kenya	Asembo	221	0	0	January - March, 2009

Two reference strains (B18962, and B37080) that have been identified as *B. bovis* were included in the MLST analysis. B18962 is the subculture of strain 91-4^T^ (the type strain of *B. bovis*) described in a cow in France [21], and B37080 is an isolate from a cow with endocarditis in the USA [27].

### Bartonella culturing

Animal bloods were thawed at 4° C and re-suspended 1:4 in brain heart infusion broth supplemented with 5% amphotericin B (to reduce likelihood that bacterial and fungal contaminants would overgrow the overgrow the slow-growing bartonella bacteria), then plated on heart infusion agar containing 10% rabbit blood and incubated in an aerobic atmosphere with 5% carbon dioxide at 35°C up to four weeks. Bacterial growth was monitored at the end of each week. Bacterial colonies were presumptively identified as bartonella based on colony morphology. Subcultures of bartonella colonies from the original agar plate were streaked onto secondary agar plates and incubated at the same conditions until sufficient growth was observed, usually between 5-7 days. Pure cultures were harvested in 10% glycerol. 

### DNA preparation, PCR verification and species differentiation by *gltA*

We extracted genomic DNA from pure culture of each isolate using the QIAxtractor automated DNA purification system (Qiagen, Valencia, CA). We performed PCR using primers 430F and 1210R to amplify a specific region in the citrate synthase gene (*gltA*) of the genus of *Bartonella* for verification of *Bartonella* strains. Positive PCR products were sequenced using the same primers as the initial PCR assay at a concentration of 1.6 µM. Using Lasergene software package (DNASTAR, Madison, WI), the *gltA* sequences obtained from all samples in the present study were compared with *B. bovis*, other ruminant-associated *Bartonella*, and other known *Bartonella* spp. for species identification.

### MLST analysis

Strains identified as *B. bovis* based on *gltA* sequences were further analyzed using MLST for eight additional loci (16S rRNA, *ftsZ*, *groEL*, *nuoG*, *ribC*, *rpoB*, *ssrA*, and ITS). Primers and other related information are provided in Table 2. We randomly selected one to three strains from each region of a country where we found bartonella to be present in the animals. Including the two reference strains from France and USA, a total of 28 strains were characterized for the MLST analysis (Table 3). A neighbor-joining tree based on the concatenated MLST alleles was constructed using the Clustal W program within MegAlign of the Lasergene package. 

**Table 2:** Characteristics of the nine loci evaluated for the *B. bovis* MLST scheme.

Locus	Forward primer	Reverse primer	length of analyzed sequence (bp)	No. variable sites	No. alleles
16SrRNA	caggcctaacacatgcaagtc	gggcggwgtgtacaaggc	1172	0	1
*ftsZ*	attaatctgcaycggccaga	acvgadacacgaataacacc	885	20	7
*gltA*	gctatgtctgcattctatca	gat cyt caa tca ttt ctt tcc a	753	18	5
*groEL*	gaactngaagataagttngaa	aatccattccgcccattc	1081	27	12
*nuoG*	ggcgtgattgttctcgtta	cacgaccacggctatcaat	328	18	4
*ribC*	taaccgatattggttgtgttgaag’	taaagctagaaagtctggcaacataacg	535	18	8
*rpoB*	cgcattggcttacttcgtatg	gtagactgattagaacgctg	852	12	8
*ssrA*	gctatggtaataaatggacaatgaaataa	gcttctgttgccaggtg	287	4	5
ITS	cttcagatgatgatcccaagccttctggcg	gaaccgacgaccccctgcttgcaaag a	364-398	48	7

**Table 3:** Allelic profiles, sequence types (ST), and lineage group (LG) classification for the 29 *B. bovis* isoaltes from different geographic locations and bovine hosts.

#	Isolate	16S rRNA	*ftsZ*	*gltA*	*groEL*	*nuoG*	*ribC*	*rpoB*	*ssrA*	ITS	ST	LG	Country	Host
1	B18962	1	1	1	1	1	1	1	1	1	ST1	1	France	cattle
2	B37080	1	1	1	2	1	1	1	1	1	ST2	1	US	cattle
3	B33663	1	1	1	3	1	1	1	1	2	ST3	1	Guatemala	cattle
4	B38041	1	1	1	3	1	1	1	1	2	ST3	1	Guatemala	cattle
5	B33664	1	1	1	2	1	1	2	1	3	ST4	1	Guatemala	cattle
6	B38038	1	1	1	4	1	1	3	1	3	ST5	1	Guatemala	cattle
7	B33695	1	2	2	5	2	1	5	1	1	ST6	2	Guatemala	cattle
8	B38216	1	2	2	5	2	2	5	1	4	ST7	2	Guatemala	cattle
9	B38241	1	2	2	5	2	2	5	1	4	ST7	2	Guatemala	cattle
10	B38035	1	3	1	6	1	4	6	1	1	ST8	1	Guatemala	cattle
11	B38215	1	3	1	7	1	5	3	1	5	ST9	1	Guatemala	cattle
12	B38223	1	4	1	1	3	1	6	1	6	ST10	1	Guatemala	cattle
13	B38240	1	5	3	6	3	1	3	1	1	ST11	1	Guatemala	cattle
14	B31166	1	4	1	1	3	1	6	1	1	ST12	1	Georgia	cattle
15	B31219	1	4	1	1	3	1	6	1	1	ST12	1	Georgia	cattle
16	B31170	1	4	1	1	3	6	6	1	1	ST13	1	Georgia	cattle
17	B31158	1	4	1	8	3	6	6	1	1	ST14	1	Georgia	cattle
18	B31167	1	4	1	8	3	6	6	1	1	ST14	1	Georgia	cattle
19	B31178	1	5	1	1	3	1	2	3	1	ST15	1	Georgia	cattle
20	B31182	1	5	1	9	1	1	6	1	1	ST16	1	Georgia	cattle
21	B25099	1	2	2	5	1	2	4	2	4	ST17	2	Thailand	cattle
22	B25093	1	2	2	5	2	2	4	2	4	ST18	2	Thailand	cattle
23	B25100	1	2	2	5	2	3	4	2	4	ST19	2	Thailand	cattle
24	B32674	1	6	4	10	4	7	7	4	7	ST20	3	Thailand	water buffalo
25	B32780	1	6	4	10	4	7	7	4	7	ST20	3	Thailand	water buffalo
26	B32850	1	6	4	10	4	7	7	4	7	ST20	3	Thailand	water buffalo
27	B32781	1	6	4	11	4	7	7	4	7	ST21	3	Thailand	water buffalo
28	B32730	1	7	5	12	4	8	8	5	7	ST22	3	Thailand	water buffalo

Newly-identified alleles from the current study were submitted to GenBank with the following accession numbers: KF193407 - KF193413, KF199895 - KF199899, KF212449 - KF212460, KF218206 - KF218209, KF218210 - KF218216, KF218217 - KF218224, KF218225 - KF218229, and KF218230 - KF218236 for *ftsZ*, *gltA*, *groEL*, *nuoG*, *ribC*, *rpoB*, *ssrA*, and ITS, respectively.

## Results

### Bartonella prevalence

Seven bartonella isolates were obtained from water buffaloes from Thailand, giving an apparent prevalence of 6.8% (7/103). In cattle, a total of 175 bartonella isolates were obtained from all countries. The prevalence of bartonella varied widely across the study sites. In Thailand, bartonella was isolated from 10% (4/40) of the cattle. In Guatemala, the overall bartonella prevalence was 20.6% (80/389) with a range of 10% - 48.7% across the six departments. In Georgia, bartonella prevalence was 73% (73/100) and 90% (18/20) in cattle from Dusheti and Marneuli districts, respectively, but bacteria were absent (0/39) in cattle from Mestia district. In western Kenya and all sampled sites of Japan, bartonella was absent in cattle, despite the large number of samples investigated in both sites. Details are provided in Table 1.

### *Bartonella* spp. identification

All 182 isolates obtained from water buffaloes and cattle from the five countries in the present study were characterized by sequencing *gltA*. The *gltA* sequences showed that all seven isolates from water buffaloes and 165 isolates from cattle from Thailand, Guatemala and Georgia, were *B. bovis* with 99.1-100% identity to the type strain (AF293394); nine isolates recovered in cattle from Dusheti District of Georgia was *B. chomelii* with 98.9-100% identity to a previously described variant (AY254308) from cattle in France; and the last isolate recovered in cattle from Marneuli District of Georgia was *B. schoenbuchensis* with 97.2% similarity to the *B. schoenbuchensis* variant (AJ567635) from China.

### Allelic profiles, sequence types (ST), and phylogenetic analysis

The size of sequenced alleles ranged between 287bp - 1172bp at different loci (Table 2) with the total length of concatenated sequences 5085bp - 5119bp. Noticeably, the ITS locus varied in size among the strains, ranging between 364bp - 398bp. Specifically, sequences of most strains were 386bp long while sequences of all strains from Thai cattle and two strains from Guatemala (B38216 and B38241) were shorter (364bp) with a 21-bp deletion, and on contrary, sequences of all water buffalo-originated strains were longer (398bp) with a13-bp insertion (data not shown). Sequencing analysis demonstrated that 16S rRNA was invariant and was identical to the previously described variant (NR025121) from *B. bovis* type strain. All other loci showed large variation, with variable sites of 4 - 48, and 4 - 12 alleles by loci (Table 2).

**Figure 1:**
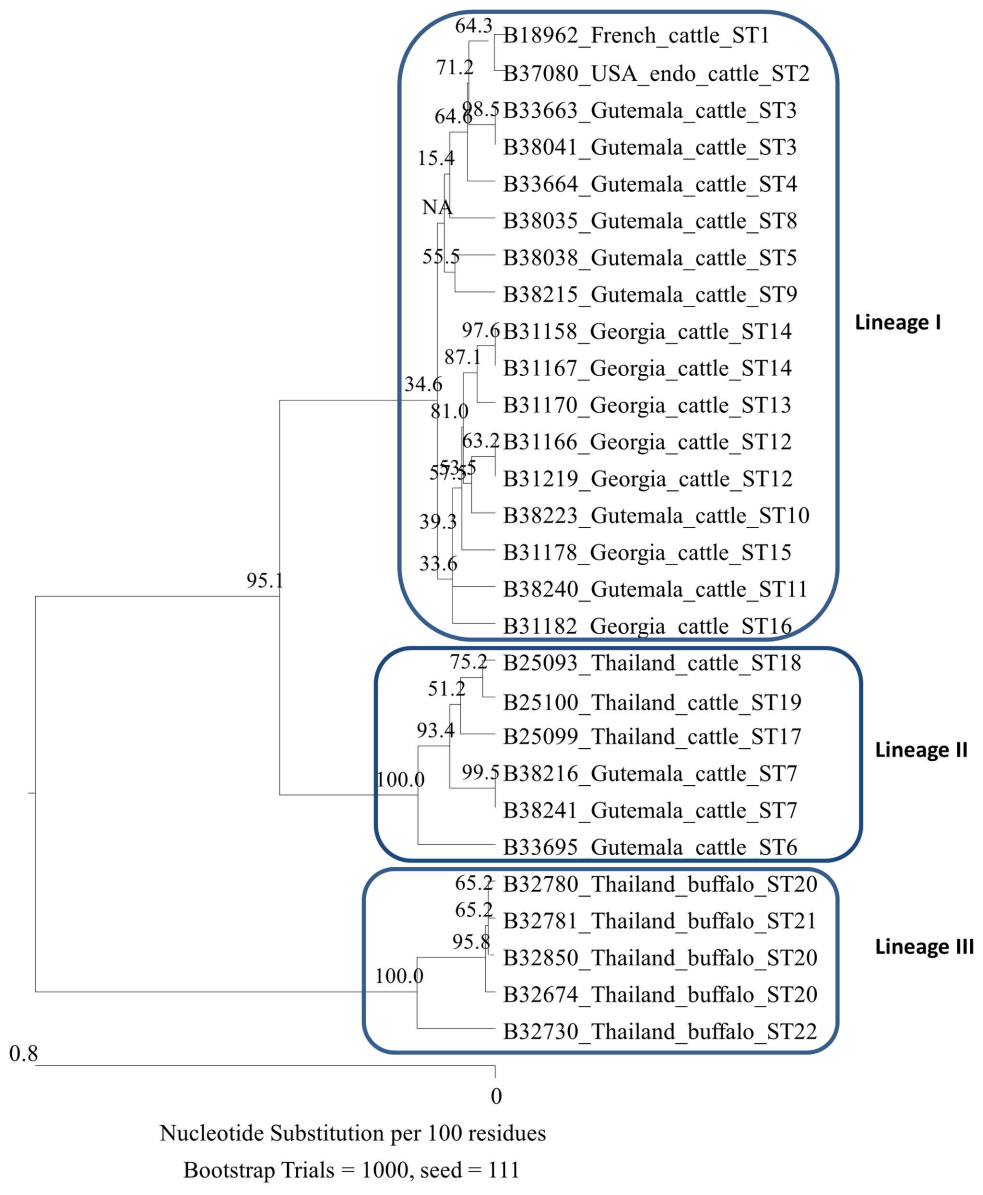
Phylogenetic relationships of the 28 *B. bovis* isolates inferred from 5085bp - 5119bp concatenated sequences of *ftsZ*, *gltA*, groEL, *nuoG*, *ribC*, *rpoB*, *ssrA*, and ITS fragments. Following each isolate are geographical origin, bovine host source, and sequence type (ST) classification. Phylogenetic tree was constructed by N-J method, and bootstrap values were calculated with 1,000 replicates. A total of 22 STs were identified, which fall into three lineages (square circled clades).

Due to the identity of 16S rRNA for all strains, the sequences of this locus were excluded from the MLST analysis. Based on the concatenated sequences of other eight loci, the MLST analysis distinguished 22 STs among the 28 strains. ST20 was represented by three strains; and ST3, ST7, ST12, and ST14 each were represented by two strains; all other 17 STs each were represented only by one strain. Phylogenetic analysis demonstrated that all STs resolved into three lineages (Figure 1, 16S rRNA not included) with low divergence (0.1 - 1.8%) among all STs. Lineage I contains 14 STs of 17 strains with divergence among the STs of 0.1 - 0.5%. Strains in this group have diverse geographical distribution, which were found in cattle from all studied regions except for Thailand; lineage II contains five STs of six strains with divergence of 0.1 - 0.4% among the STs. The strains within lineage II were obtained from cattle from Thailand (3 strains, of ST6 and ST7) and Guatemala (3 strains, of STs17 - 19); and lineage III contains five strains obtained exclusively from water buffaloes from Thailand. The five strains belonged to three STs (STs20 - 22) with divergence of 0.1 - 0.4%. The distances between lineage III and either lineage I (1.4 - 1.5%) or lineage II (1.7 - 1.8%) were much more distant comparing to that between lineages I and II (0.7 - 1.1%).

### Host association and geographical distribution of *B. bovis* sequence types

Strains from the same location exhibited different sequence types, showing little evidence of concordance between the MLST data and geographical origin. However, all sequence types showed clear host association, either with cattle or water buffaloes. Furthermore, except for Guatemalan strains, other STs obtained from the same area were more associated with a specific lineage. For example, all STs from Georgia, France, and the US belong to lineage I; STs obtained from Thai cattle belong to lineage II; while all STs within lineage III were exclusively from water buffaloes. For the 11 STs obtained from cattle from Guatemala, eight of them fall into lineage I while the other three fall into lineage II.

## Discussion

In this study, we investigated infections with *Bartonella* spp. in water buffaloes from Thailand and cattle from five countries across the world. We provided the first evidence that water buffaloes are also infected with *Bartonella* spp., with a prevalence of 7%. In cattle, bartonella prevalence varied widely between the studied countries and between different regions in the same country. In Japan, Kenya, and the Mestia district of Georgia, the cattle were free from the infection; in contrast, infection prevalence reached as high as 90% in the Marneuli district of Georgia, and a moderate prevalence was observed in other areas. It is of interest to note that the prevalence of infection in cattle varied widely across districts in Georgia, from apparent absence to nearly 100%. The observed variation in bartonella prevalence in cattle between different countries/regions can be the result of multiple factors. Since bartonellae are mainly vector-transmitted [11-16], we speculate that the distribution and abundance of specific arthropods play a major role in this matter. It may be that a heavy level of infestation is required for transmission. Cattle ticks (*Rhipicephalus microplus*) and biting flies (Diptera spp.) are implicated as potential vectors that may transmit bartonella between cattle [31,32]. Interestingly, low ectoparasite infestation on cattle in Japan was noticed (Maruyama, personal communication). Such a fact could provide a plausible explanation for the absence of bartonella infection observed in cattle from Japan. In addition to ectoparasites, environmental factors, such as geographic characters, landscape, etc., may also have influences on bartonella prevalence. Of the three districts in Georgia, Mestia district (with no bartonella infection in cattle) is located in a mountainous area in the northwest of the country, while the other two districts (with high bartonella infection in cattle) are located in lower-lying areas in the northeast and southeast. 

The cultures obtained from the study belonged to three *Bartonella* spp. based on *gltA* sequences. The majority of strains (172/182), identified as *B. bovis*, were the most common in cattle from all investigated places. This is concordant with previously reported observations. The other two species, *B. chomelii* and *B. schoenbuchensis*, are not common, although both have been previously reported in cattle [17,25]. Interestingly, both of these species were only found in cattle from Georgia. This may suggest that the bartonella community associated with cattle in Georgia is more diverse comparing to other places. All nine *B. chomelii* isolates were obtained from cattle in Dusheti District located in northeastern Georgia. These observations may be associated with the composition of the local ectoparasite community. Future studies of cattle ectoparasites should test hypotheses about whether any particular arthropod species act as vectors for bartonella transmission between cattle. Finally, all isolates obtained from water buffaloes in Thailand also belong to *B. bovis*, indicating that water buffaloes can also serve as hosts for *B. bovis*.

 Although *B. bovis* is known to be widely distributed in bovines, no study has demonstrated how strains from different geographic areas and hosts vary. As a potential pathogen for domestic animals, information about genetic diversity of *B. bovis* is important. In this study, we developed an MLST scheme for *B. bovis* based on nine loci to characterize *B. bovis* strains among geographically diverse populations. Resolving into 22 STs among the 28 strains from different geographical regions, our MLST data demonstrated *B. bovis* strains can be genetically different. Although some STs can be quite close, the same ST was never found in strains from different regions. 

Multi-locus sequencing typing has been applied in a number of studies previously, in which the MLST was based on the comparison of multiple housekeeping gene sequences [28-30,33]. In this study, we included ITS. Although non-functional, sequence comparison of the ITS region is widely used in taxonomy and molecular phylogeny for its ease of amplification and high degree of variation, even between closely-related species. Our data showed either insertion or deletion has occurred in some of the tested strains, which likely are associated with host species. Such results suggested that ITS might be more powerful in differentiating genetic diversities among strain populations, compared to some regularly-used housekeeping genes. 

Identification of three close but distinct lineages among the STs suggests a clonal population structure for the species. All STs of cattle-originated strains fall into either lineage I or II, while STs of water buffalo-originated strains exclusively fall in lineage III, showing the specific host relationship. Meanwhile, we suspect that lineage I and lineage II each is associated with a particular lineage of *Bos taurus*. By general understanding, cattle from US, Europe, and Georgia belong to the ‘taurine’ lineage (former *B. taurus* subsp. *taurus*); while those from Thailand belong to the ‘zebu’ lineage (former *B. taurus* subsp. *indicus*); and in Guatemala, cattle were of mixed breeds. Based on this information, we hypothesize that lineage I is associated with cattle of ‘taurine’ lineage and lineage II is associated with cattle of ‘zebu’ lineage. Further studies are required to confirm this hypothesis. 

MLST analysis also showed *B. bovis* strains retain geographical particularity. This observation can be explained by the association of *B. bovis* with specific breed of cattle as well. For example, all cattle-originated strains from Thailand belong to lineage II; while all strains from Georgian cattle together with strains from the US and France, belong to lineage I. Contrastingly, strains from Guatemalan cattle fall into both lineages I and II. Mixed breed of cattle in Guatemala likely is the cause of crossing lineages. 

## Conclusion

Lineage I was the most common group of *B. bovis*, containing most STs (13/22) that are distributed in different regions across the world. The isolate that caused cattle endocarditis in the US (B37080, ST2) was also within this lineage. Although none of the other tested strains is of the same ST, all STs in this lineage are in fact very close. While clinical symptoms were not recorded for cattle in these studies, *B. bovis* strains have high potential as a pathogen, being widely distributed in cattle populations globally.
